# *Caenorhabditis* Intervention Testing Program: the anticonvulsant levetiracetam does not extend lifespan in nematodes.

**DOI:** 10.17912/micropub.biology.001987

**Published:** 2026-03-31

**Authors:** Jyothi Shilpa Akella, Brian D Onken, Christine A Sedore, Yuhua Song, Vijaya Madhuri Achanta, Monique Walker, Jian Xue, Suzhen Guo, Anna Coleman-Hulbert, Patrick C Phillips, Gordon J Lithgow, Monica Driscoll

**Affiliations:** 1 Department of Molecular Biology and Biochemistry, Rutgers University, Piscataway, New Jersey 08854, USA; 2 Institute of Ecology and Evolution, University of Oregon, Eugene, Oregon 97403, USA; 3 The Buck Institute for Research on Aging, Novato, California 94945, USA

## Abstract

The *Caenorhabditis* Intervention Testing Program aims to identify lifespan-extending interventions that are effective across diverse genetic backgrounds. Previous studies identified a role for anticonvulsants in lifespan extension in the nematode *Caenorhabditis elegans* . The FDA-approved anticonvulsant levetiracetam acts in *C. elegans* and modulates neurotransmission. However, levetiracetam effects on nematode lifespan are unknown. Here, we examine the effect of levetiracetam on the lifespan of strain representatives from three species of the *Caenorhabditis* genus. Our results do not reveal an effect of levetiracetam on nematode lifespan, indicating that anticonvulsants can differ in their ability to extend lifespan.

**Figure 1. Levetiracetam does not offer lifespan benefits in some CITP strain backgrounds:** Survival curves for *C. elegans* N2 , *C. briggsae* ED3092 , and *C. tropicalis* JU1373 treated with a range of levetiracetam concentrations starting on day 1 of adulthood. Each line represents lifespan data of pooled replicates from a minimum of two trials. Levetiracetam had no effect on lifespan at any concentration in all the backgrounds tested here. P = not significant for all concentrations. Statistical comparisons were performed using the Cox proportional hazards (CPH) model with mixed effects using the coxme package (v2.2-22) in R (v4.3.3) (Therneau, 2024; R Core Team, 2025). We used a hierarchical randomized block design estimated via a restricted maximum likelihood general linear model using the lme4 package (1.1-35-5) to assess variance components for longevity within each strain: Trial was responsible for 9.7-12.8% of total variance, experimenters within a trial accounted for 1.6-4.9% of total variance, and 0.3-2.8% of variance was attributable to individual plates within an experimenter, in line with previous CITP publications (Lucanic et al., 2017; Driscoll et al., 2025).

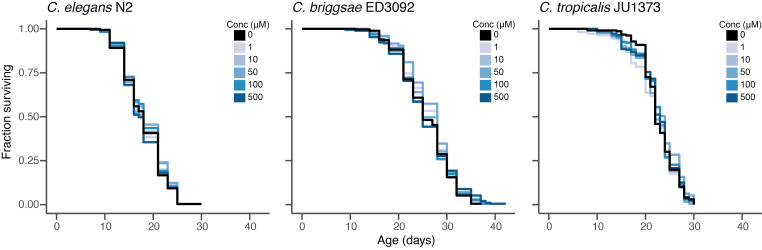



## Description

The *Caenorhabditis* Intervention Testing Program (CITP) is a National Institute of Aging-funded multi-institutional consortium that aims to identify compounds that robustly increase lifespan across genetically diverse populations (Driscoll et al., 2025). The compounds that are chosen for testing in the CITP pipeline can be selected based on previously identified or predicted links to lifespan/healthspan extension. One class of compounds that has been identified to have a strong effect on lifespan is anticonvulsants. Previous studies have shown that anticonvulsants such as ethosuximide and sodium valproic acid lead to increases in lifespan in the N2 reference strain of *Caenorhabditis elegans* (Evason et al., 2005; Evason et al., 2008). Levetiracetam is an anticonvulsant approved by the FDA as a treatment for seizures. In *C. elegans ,* levetiracetam is known to play a role in modulating neurotransmission: levetiracetam rescues the extended paralysis observed in an electrical shock-treated loss-of-function mutant of GABA receptor encoding gene *unc-49* (Risley et al., 2016). Despite previous studies linking anticonvulsants to lifespan extension, whether levetiracetam plays a role in lifespan extension remains unknown.

&nbsp;

We tested survival of *C. elegans* N2 , *C. briggsae* ED3092 , and *C. tropicalis* JU1373 at levetiracetam doses ranging from 0 to 500 µM (we saw deleterious effects in both N2 and ED3092 at the 500 µM dose, suggesting toxicity above this concentration). We did not observe enhanced survival under any treatment within this dosage range (Figure 1). Our results may reflect the inability of levetiracetam to engage lifespan regulation in diverse *Caenorhabditis* strains. Previous studies with levetiracetam were conducted at high doses, brief exposure (Risley et al., 2016). Poor compound uptake, compound stability issues, bacterial metabolism of the compound, or failure to test a critical dose of the compound that could have an effect on lifespan might equally explain the absence of lifespan modulation that we observed in this study. However, the CITP charge is to test compounds under standardized CITP assay conditions, multiple backgrounds, multiple sites, multiple doses. When compounds are not efficacious, we cannot extend efforts to discern why a compound did not work. Future experiments testing for rescue of electroshock-induced paralysis in *unc-49* mutants by levetiracetam treatment at tested doses, or examining effects of levetiracetam on locomotion and pharyngeal pumping, could speak to issues with compound bioavailability and dosage.

&nbsp;

In sum, our data show that levetiracetam does not robustly influence lifespan under experimental conditions employed by the CITP. Previous CITP analysis of valproic acid did not reveal lifespan extension (Lucanic et al., 2017), but we have pointed out that CITP studies are conducted under different exposure conditions than used in the positive outcome trials (Evason et al., 2005; Evason et al., 2008). Anticonvulsants may have to engage *C. elegans* physiology under precise conditions with narrow windows of impact to promote longevity. Alternatively, it is plausible that not all anticonvulsants will confer survival benefits.

## Methods

Here we examined the effects of levetiracetam on the lifespan of *C.* *elegans* and other strain representatives of CITP species following our previously published protocols (Lucanic et al., 2017). The CITP uses adult treatment to better mimic the first likely exposure window in the clinic and to exclude any possible developmental defects due to larval application of compound. Briefly, animals were first synchronized using a timed egg lay, in which adult animals were allowed to lay eggs for four hours on nematode growth media (NGM) plates seeded with *E. coli* OP50-1 , after which the adults were picked off the plate, leaving tightly synchronized eggs that were grown to adulthood. Fifty day 1 adult animals were transferred in triplicate onto OP50-1-seeded 35 mm NGM agar plates that also contained 51 µM 5-Fluoro-2'-deoxyuridine. A levetiracetam stock solution (Sigma) was prepared in water at a concentration that allowed 125 µL of the levetiracetam solution to be added to each 3 mL NGM plate to achieve final concentrations of 1, 10, 50, 100, and 500 µM. Control plates were treated with 125 µL of water. Animals were maintained at 20˚C and 80% relative humidity then moved to fresh plates with levetiracetam on days 1, 2, and 4 ( *C. tropicalis )* or 5 ( *C. briggsae* and *C. elegans* ) of adulthood, and once per week after that, for lifetime. Animals were scored manually for movement thrice weekly and those that did not move spontaneously or after gentle prodding with a 0.2 mm diameter platinum wire were considered dead. Animals with internal hatching of embryos, extrusions, or those who walled, burrowed, or otherwise escaped the NGM plate were censored in the final analysis. The experiment was repeated with at least two biological replicates and a total of 220-290 animals per strain and concentration. Raw data and statistical summaries can be found on the CITP Data Portal (CITPaging.org/portal, version 3.0).

## Reagents

The following strains were obtained from the *Caenorhabditis* Genetic Center (CGC), which is funded by the NIH Office of Research Infrastructure Programs (P40 OD010440): *C. elegans* N2- PD1073 , *C. briggsae* ED3092 , and *C. tropicalis* JU1373 . Levetiracetam was obtained from Sigma (Levetiracetam L8668).
